# Cannabis use during pregnancy and its relationship with fetal developmental outcomes and psychiatric disorders. A systematic review

**DOI:** 10.1186/s12978-020-0880-9

**Published:** 2020-02-17

**Authors:** Carlos Roncero, Isabel Valriberas-Herrero, Marcela Mezzatesta-Gava, José L. Villegas, Lourdes Aguilar, Lara Grau-López

**Affiliations:** 1 Psychiatric Service, University of Salamanca Health Care Complex, Salamanca, Spain; 2 Institute of Biomedicine of Salamanca (IBSAL), University of Salamanca, Salamanca, Spain; 3 Multidisciplinary Unit of Autism Spectrum Disorder (UnimTEA), Mental Health Department, Sant Joan de Deu Hospital, Barcelona, Spain; 4 Addiction and Dual Diagnosis Unit, Psychiatric Service, Vall d’Hebron University Hospital, CIBERSAM, Barcelona, Spain; 5 Group of Psychiatry, Mental Health and Addiction, Vall d’Hebron Research Institute, Barcelona, Spain; 6 Department of Psychiatry and Forensic Medicine, Autonomus University of Barcelona, Barcelona, Spain

**Keywords:** Cannabis, Mental disorders, Pregnancy, Prenatal Cannabis exposure, Risk factors, Prenatal marijuana exposure

## Abstract

**Introduction:**

This study analyze factors associated to cannabis use in pregnant women, its perceived availability, its risk perception and the relationship between prenatal exposure to cannabis and developmental and mental disorders.

**Objectives:**

We present a review of the literature on cannabis use among pregnant women. The objective is to analyze factors associated to cannabis use during pregnancy and assess the potential effects of prenatal exposure to cannabis on the development of the fetus and the mental health of those exposed.

**Methods:**

Systematic review of studies on the maternal use of cannabis and the relationship between early exposure and the development of psychiatric disorders in the PubMed database until July 2018 in English and Spanish with the following keywords: Marijuana, Cannabinoids, Mental disorders, Pregnancy, Prenatal Cannabis Exposure, Risk factors.

**Results:**

The use of cannabis among pregnant women is frequent but it has not been extensively researched. Prenatal exposure to cannabis may be associated with affective symptoms and ADHD.

**Conclusions:**

Mental healthcare professionals who treat women during their fertile life need to be able to explain the relationship between prenatal exposure to cannabis and the presence of developmental and mental disorders.

## Plain English summary

Cannabis is the most consumed illegal drug in Europe. Substance abuse in pregnant women has increased over the past decades. Cannabis use during pregnancy is underdetected.

The objective of this review is to analyze factors associated to cannabis use during pregnancy and assess the potential effects of prenatal exposure to cannabis on the development of the fetus and the mental health of those exposed.

After conducting a search on the main online database and studying the available literature, we found that cannabis use during pregnancy may be associated with abnormalities in general development, as well as with changes in brain chemistry both in humans and in research animals. Furthermore, exposure to cannabis during pregnancy may be associated with the development of mental disorders such as Attention Deficit Hyperactivity Disorder (ADHD) and depression.

Early detection and alerting pregnant women about the risks of cannabis use during pregnancy is one way to minimize its possible harm. Therefore, we need to expand our scientific knowledge and to train health professionals in this field.

## Introduction

Cannabis is the most widely used illegal drug in Spain and in Europe [1]. According to the EMCDDA (European Monitoring Centre for Drugs and Drug Addiction), 26.3% of all Europeans have tried cannabis throughout their lives and 7.2% have used it in the last year, with a prevalence of use at 14.1% among young adults between 18 and 35 years [1].

In Spain and most of Europe, cannabis continues to be an illegal substance, however, there are some regions in the western world where this has changed as some states of United States. Recently, other countries like Uruguay, or more recently, Canada have legalized their consumption [2].

In Spain, the average age of first cannabis use is 18.3 years [2], although the main age group is the population of ages 15 to 24, in which 19.9% reports having used cannabis in the last 12 months [2]. This finding is in line with the European School Survey Project on Alcohol and Other Drugs (ESPAD), which includes information on substance use among 15- to 16-year-old students in 35 European countries. In the group of 24 Member States of the EU plus Norway, 18% of the students reported having tried cannabis at least once (lifetime prevalence); the highest levels were observed in the Czech Republic (37%) and France (31%); and 8% said that they had used cannabis in the last month [1].

In the 14–18 age group, the Secondary School Survey on Drugs in Spain (ESTUDES, 1994–2016) [2] was carried out, and it confirmed that the most commonly used illegal substance in Spain is cannabis, with an average age of 14.9 years for first use. Given its prevalence among adolescents and young adults, we can say that the use of cannabis has a large impact nowadays. In addition, the risk perception among the young population is lower than with other drugs [2]. Young people have a low perception of the risk associated to the use of cannabis, in spite of all the available evidence about its physical, psychological and social consequences. Cannabis is the most widely available psychoactive substance after alcohol and tobacco [2].

Differences are observed in substance use between men and women [2]. The use of legal drugs is more widespread among women and the use of all illegal drugs is more prevalent among men. The differences in prevalence based on gender decrease with decreasing age, since in the population aged 14–18 almost no differences regarding gender were found [2]. However, the gender inequality decreased in the 1996–2009 period [1], and all the prevalence indexes increased for women [2], particularly young women of fertile age.

There are very few data on pregnant women that let us deduce the real prevalence of use; however, the analysis of the meconium of newborns from mothers who gave birth in Spanish public hospitals revealed cannabis in 5.3% of the cases [3], a figure which is similar to what was found in other international studies (4.5% of all pregnancies).

Volkow describes that in the US between 2002 and 2003 and 2016–2017, adjusted prevalence of past month cannabis use increased from 3.4 to 7.0% among pregnant women [4]. Cannabis was the illegal drug most commonly consumed by pregnant women in western countries [3, 5, 6].

However, there are some challenges in cannabis detection, derived from under-reporting, the fear of legal consequences, the possible loss of the children’s custody, and the feelings of guilt caused by the potential effects on the baby.

The use of cannabis in pregnancy is very relevant for its effects on the development of the fetus may be subtle at first and not be detectable for many months to years after birth, but the physical and psychopathological consequences on the adult life may be severe. Evidence on these effects is plentiful but ambiguous [5].

For this reason, the objective of this study is to review the literature on the use of cannabis among pregnant women, its associated factors and its potential effects on the development of the fetus during the postnatal period, childhood and adolescence.

## Material and methods

A search in English and Spanish was carried out on the PubMed database for matches until July 2018 with the following keywords: “Marijuana”, “Cannabis”, “Cannabinoids”, “Mental disorders”, “Pregnancy”, “Prenatal Cannabis Exposure”, “Risk factors”, “Prenatal Marijuana Exposure”. The keywords were combined as follows:

“Prenatal Cannabis exposure”; “Prenatal Marijuana exposure”; “Prenatal Cannabis exposure” AND Pregnancy AND Marijuana AND Cannabinoids; “Prenatal Cannabis exposure” AND Pregnancy AND Marijuana AND “Mental disorders”; “Prenatal Cannabis exposure” AND Pregnancy AND Marijuana AND “Risk factors”; Pregnancy AND Cannabis.

The inclusion criteria were: studies specifically focused on associated factors of cannabis use during pregnancy, effects of exposure to cannabis on the developing fetus and its mental health.

**Fig. 1:**
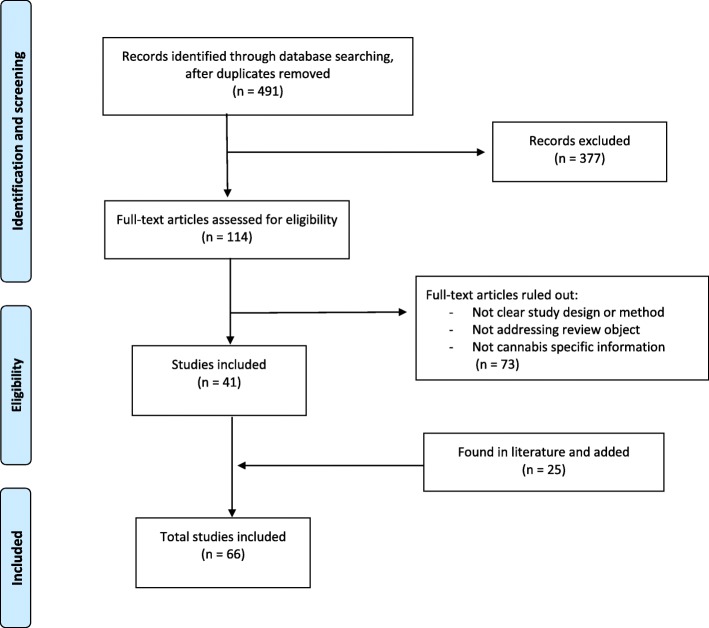
Review Flowchart

The search revealed 491 potentially adequate articles, 377 of which did not meet the inclusion criteria after a review of their title and their abstract. One hundred fourteen articles were selected, 73 of which were ruled out after a full reading. Ultimately, 41 articles from the original search were included [3, 5–44], plus 25 articles which were added after appearing repeatedly in the list of references of the first group [1, 2, 4, 45–66]. According to the PRISMA methodology [63], 73 articles were ruled out because of a small sample size, unclear designs or methods of study, not addressing review objectives or not specific information about cannabis. Fig. 1.

The review includes 4 articles that focus on the prevalence of prenatal exposure to cannabis [3, 5, 6, 45], 7 articles that discuss detection methods for the use of drugs in pregnant women [7–12, 46], 9 molecular studies [13–20, 47], 6 studies on rodents [21–25, 48] and 33 articles on the relationship between cannabis exposure and developmental/psychopathological consequences [26–44, 49–62].

## Results

This section describes the results of the review of articles on associated factors to cannabis use during pregnancy and on the prenatal exposure to cannabis and its possible relationship with developmental disorders and/or psychopathological consequences.

### Factors associated to cannabis use during pregnancy

Heterogeneous results have been obtained from different studies, this is probably related to differences in sample populations, study designs used and cultural differences from the geographical locations in which these studies are carried out.

El Marroun et al. can not find any strong association with demographic characteristics as age, ethnicity or presence of psychopathology with cannabis use during pregnancy in the study he performed in Rotterdam. But it is described a strong association with biological father’s cannabis use and being unmarried. Religion is described as a protective factor. From this sample 3′2% of women used cannabis before being pregnant, 2′9% before and during pregnancy, but just 0′6% of women decided to continue cannabis use throughout pregnancy. This last group had a lower educational level [6].

They also find out that history of cannabis addiction makes 2.77 times more likely to continue cannabis use during pregnancy; also, women with a frequent cannabis use (daily or weekly) are more likely to continue it than those who use it monthly [6].

However, Gray et al., in a study performed in US, cannot describe strong association with demographic characteristics as age, being unmarried or being employed. It is described that Hispanic women are less likely to use cannabis during pregnancy, but cannabis use was more likely in women from multiracial origin [9]. This differs from another study conducted in the US in which an association is found between cannabis use during pregnancy and characteristics such as being young, unmarried and non-Hispanic white. It is associated with having a psychiatric disease different from substance addiction and not having graduated from high school [65].

The knowledge of these associated factors to cannabis use during pregnancy may be useful in order to identify future mothers to provide with quality information about the possible consequences of prenatal exposure to cannabis [66].

### Animal studies on the effects on children of prenatal cannabis exposure

In animal models, controlled doses of cannabinoids were administered to pregnant or very young animals. Afterwards, the studies assessed the effects on the development of the CNS, the neurotransmission systems, the appearance or enhancement of drug-seeking behavior, the presence of altered behavior and the psychomotor skills, in order to infer the presence of the equivalent to “mental disorders” in animals [21–25, 48]. The use of cannabis in rats causes changes in the dopaminergic activity of the *corpus striatum* which leads to attention deficit and hyperactivity disorders and alterations in locomotion [21], and on the *prefrontal cortex*, which causes cognitive impairment and emotional dysregulation. Prenatal exposure in rodents causes an increased rate of ultrasonic vocalizations when separated from the mother, which leads to increased levels of anxiety that are related to the presence of CB1 receptors in the cortex, the hippocampus, the lateral septum, the nucleus accumbens and the amygdala, which regulate the release of 5-HT, dopamine, CCK and CRF, which are anxiogenic peptides [13, 21].

In addition, changes take place in the dopaminergic activity of the hypothalamic–pituitary axis and the amygdala, which are involved in emotion regulation [13]. It has been observed that the exposure of rats to low or moderate doses of the cannabinoid agonist WIN 55,212–2 causes permanent alterations in the cortical glutamatergic system and affects the migration of glutamatergic neurons and GABAergic interneurons [22, 23]. Exposure to this agonist induces alterations in the intrinsic electrophysiological properties of the Purkinje neurons of the cerebellum and causes alterations in the motor and exploratory activity [24]. Alterations of endorphins and an enhancement of opioid-seeking behavior have been described mainly in female rats [21, 48]. However, there is controversy as to whether exposure to THC in adolescent animals alters opioid reinforcement in the adult life [26] and increases the self-administration of heroin [26, 48] (Table 1). The evidence suggests that, in animals, there are persistent changes after the use of cannabis regarding behavior, motivation, the reinforcement caused by drugs and the response to stress [48].

**Table 1:** Described consequences of Perinatal Cannabis Exposure in Humans and Rodents

	Humans	Rodents
System	*- alterations on the mesocorticolimbic system* [15, 16]. - *thicker prefrontal cortex* [43].	*- changes in the dopaminergic activity of the corpus striatum and on the prefrontal cortex* [21]. *- dopaminergic activity of the hypothalamic–pituitary axis and the amygdala* [13]. *- alterations in the cortical glutamatergic system and affects the migration of glutamatergic neurons and GABAergic interneurons* [22, 23]. *- alterations in the intrinsic electrophysiological properties of the Purkinje neurons of the cerebellum* [24].
Molecular	*- high CB1 mRNA expression in the fetal hippocampus and amygdala* [15]. *- decrease in the expression of proenkephalin mRNA in the fetal striatum with a dose-dependent effect* [20, 47]. *- up-regulation of proenkephalin mRNA in the mesolimbic area in adult life* [20, 47]. *- increased expression of u-opioids in the amygdala* [17–20, 47]. *- decrease of k-opioid receptor mRNA in the mid-dorsal thalamus* [17–20, 47].	

### Human studies on the effects on children of prenatal cannabis exposure’

The mechanisms through which cannabis affects the brain of the human fetus and causes neurochemical and neuroanatomical changes are not well known. Cannabinoid receptors are present in the placenta and they appear in the fetal brain at 14 weeks after conception and increase in density throughout the third trimester. Given its lipophilic nature, one third of the THC in the maternal plasma crosses the placenta and it can be excreted into breastmilk. Cannabis could alter the function of fetal cannabinoid receptors [14, 26] and it may lead to changes in the dopaminergic and opioid system [15, 16].

There is a high density of cannabinoid receptors in the frontal lobe and the cerebellum, and these structures show a parallel and late maturation when compared with other structures of the brain [25]. Studies on the exposure of human fetuses to cannabis have reported alterations and a different impact on the mesocorticolimbic system, which is in charge of the regulation of emotions [15, 16]. The CB1 receptor has been associated with the dopaminergic and opioid systems (neuropeptidase) [15], and it has been hypothesized that an increase of its expression in the hippocampus and amygdala of the fetus may suggest that these structures are more vulnerable to prenatal exposure to cannabis [15].

Intrauterine exposure to cannabis may causes different pattern of the anatomical organization of the CB1 mRNA expression in the mid-gestation fetal and adult human brain; has been found high CB1 mRNA expression in the fetal hippocampus and amygdala [15], a decrease in the expression of proenkephalin mRNA in the fetal striatum with a dose-dependent effect [20, 47], and an up-regulation of proenkephalin mRNA in the mesolimbic area in adult life [20, 47]. There is also an increased expression of *u*-opioids in the amygdala and a decrease of *k*-opioid receptor mRNA in the mid-dorsal thalamus [17–20, 47]. This all suggests that in utero exposure to cannabis fundamentally changes the systems that regulate the emotional life, such as the mesocorticolimbic pathway, and these changes may even be persistent in this individuals [17, 20, 47].

There is one study that assess the different consequences of the maternal use of cannabis in the newborn [9]. From a somatic perspective, the use of cannabis has been associated, both in the early and the late stages of pregnancy, with a higher risk of restricted fetal growth [9], low birth weight, a shorter birth length and a low head circumference [18, 27, 58, 59], hypertelorism, and epicanthus [9, 56]. From a cardiovascular perspective, an association has been observed with ventricular septal defect [28]. The in utero exposure to cannabinoids may result in a dysfunction of the T lymphocytes and a decreased immune response to viral antigens. These effects may be mediated by epigenetic mechanisms such as alterations of micro RNA, DNA methylation and modification of histone profiles. Therefore, prenatal exposure to cannabis may cause epigenetic changes that could have consequences on the later development or even long-term transgenerational effects [29]. In children who were exposed to cannabis in the prenatal stage, a thicker prefrontal cortex was observed when compared with non-exposed children [43] (Table 1).

From a psychiatric perspective, cannabis withdrawal syndrome has been described in newborns [9], although other authors have been unable to prove the existence of negative perinatal effects in children whose mothers used cannabis [58].

There are few review studies suggesting prenatal exposure to cannabis may be associated with mood and behavioral alterations, that could be related with affective mental disorders, and depressive symptoms, as well as ADHD [19, 31–34]. There are no studies that establish a connection with the presence of psychotic disorders [35].

There are longitudinal studies which describe behavioral and cognitive disorders associated with uterine exposure to cannabis, such as the *Ottawa Prenatal Prospective Study* [60] and the *Maternal Health Practices and Child Development* Project [36]. These studies did not find significant behavioral alterations in the newborns whose mothers used cannabis, but they observed that they sleep fewer hours and show habituation deficit to visual stimuli [36, 37, 61]. At age two, no cognitive alterations have been found on those children [38]. At age three, an alteration of short-term memory has been observed on verbal and abstract reasoning and on verbal skills, without any effect on intelligence [38].

The study carried out by Day et al. (2011) describes the association between prenatal exposure to cannabis and a low IQ during the school age [39]. An alteration of the executive functions regulated by the prefrontal cortex has also been described [32], including visual-spatial reasoning, response inhibition and working memory, which may last until ages 13–16 [32]. At these ages, the children may present higher impulsiveness and hyperactivity, lower attention capacity and a higher prevalence of delinquent behavior, which could be partially related to the alterations mentioned above [39]. Have been observed in two studies that the executive dysfunction could be present even into early adulthood [37, 40]. On the other hand, an increase in the use of cannabis and nicotine at ages 14–21 has been observed among individuals who had been exposed during the prenatal stage, particularly in men [37, 40]. Another longitudinal study, *The Generation R Study* [62], still ongoing, has observed an association between the maternal use of cannabis and an increase in aggressive behavior and attention disorders in girls at 18 months of age, and this association stops being statistically significant at 36 months [42, 62]. At 30 months, no differences were found in the nonverbal cognitive scales or in language development, regardless of sex [62]. Finally, a recent study establishes an association between the maternal use of alcohol and cannabis and Tourette syndrome [44].

A maternal questionnaire could not be considered an efficient screening tool for the detection of the maternal use of cannabis one of the reasons is the underreporting among pregnant women [11, 12, 46, 66], which justifies the need to use biomarkers [8]. Both the use of maternal hair and the meconium of the newborn have shown a prevalence of substance use that is higher than what was detected through clinical interviews [8].

Maternal hair provides direct information on THC use over the last months, or even years [8]. It offers a direct estimation of maternal exposure to drugs, but only an indirect estimation of the substances that reach the fetus through the placenta.

Meconium is the most widely used fetal matrix to reveal prenatal exposure to drugs of abuse [6]. It can be easily obtained, but it has a collection window of 72 h, it could be less sensitive for detection of exposure during the first trimester and testing is more expensive and less available [7, 8]. Screening of blood and urine samples during pregnancy is simpler and more readily available, but it only provides information about substance use over the last 24–48 h [10, 46].

## Discussion

There is a high prevalence of cannabis use during pregnancy, despite the difficulties to detect its consumption among pregnant women. However, it is difficult to describe factors associated to cannabis use during pregnancy due to the different results obtained in different studies. While in EU, El Marroun et al. associates being unmarried and lower education level as more likely in women that use cannabis during pregnancy, no association is found with demographic characteristics as age, employment or ethnicity [6]. In US, Martin et al. finds association with young age, not unemployed and white non-Hispanic women, this study also find association with being unmarried and having lower educational level [65]. This also differ from other US study, which finds as more likely cannabis use during pregnancy women from multiracial origins, but no differences with age, employment or marital status [9]. This heterogeneity in findings across studies could be explained with the differences in study designs used, confounding factors, sample population and sample size.

In humans, the endocannabinoid system appears at an early stage of the embryonic and fetal development, and it is related to the development of other neurotransmission systems (opioid, glutamatergic, dopaminergic, serotoninergic, etc.), which may be affected by exposure to cannabis. Prenatal exposure to cannabis could cause alterations of the activity of brain areas such as the prefrontal cortex, the mesolimbic system, the striatum and the hypothalamic-pituitary axis, which are involved in executive functions and the reinforcement and regulation of the emotional systems. So according to this hypothesis there could be neuro-cognitive consequences to the exposure, some of which may remain present even in early adulthood, such as executive dysfunction, with repercussions on the daily life [15, 16, 64].

It may exists relationship between prenatal exposure to cannabis and the presence of neurocognitive [32, 38, 40, 44] and psychiatric consequences in adult life, particularly regarding affective disorders (anxiety and depression) [13, 31, 32] and ADHD [31, 32, 34, 46], as well as emotional dysregulation, cognitive alterations and an alteration of opioid reinforcement. This last effect has been observed in studies on rodents [10, 19, 25, 31–34, 48].

It is necessary to point out that there are multiple methodological limitations in the studies in our review. We may highlight the reduced sample sizes [35]; limitations in the screening methods for the detection of substance use [11, 46], of the dose, and of the time of exposure during pregnancy; and the variability of the type and composition of the marijuana that was used [34], as well as the simultaneous use of other substances, including nicotine [9]. Similarly, there are few longitudinal prospective studies on this topic [12, 36, 60, 62]. Therefore, it is difficult to establish a clear association between exposure to cannabis during pregnancy and the potential alterations on a neurochemical and neuroanatomical level, as well as the perinatal consequences and the presence of cognitive alterations or mental disorders in the child, the adolescent and the adult individuals. However, although some limitations have been described in the different studies, we can state that the use of cannabis among pregnant women is a common occurrence, but underreported and underdetected [66]. In the studies of drug use among pregnant women, the maternal questionnaire is not an effective detection tool due to underreporting by the mothers, often caused by fear of legal consequences, fear of losing the custody of the children, or a feeling of guilt because of the potential effects on the health of the newborn. Also, due to the conditions in which the interview takes place, lack of precision regarding the moment of gestation in which they used cannabis or the amount they used [11, 12, 46]. This may justify the use of biomarkers [8].

Both maternal hair and the meconium of the newborn have shown a prevalence of substance use that is higher than what was detected through clinical interviews [8].

Hair samples have been considered the reference biological matrix to assess the chronic use of drugs during pregnancy. It involves a noninvasive procedure, a considerable amount can be obtained, and it allows a retrospective study over a longer period. Meconium samples are easy to collect and parental agreement is generally easily obtained. However, it has a short collection window (under 72 h), testing is more expensive and less available [7, 8]. Therefore, screening with blood and urine samples is used during pregnancy because it is easier and more readily available, in spite of the fact that these tests only provide information about use in the last 24–48 h [10, 46]. There are few studies using detection methods, each of them with different methods and designs. More homogeneously design studies are needed in order to develop sensitive and available methods for substance use detection.

Healthcare professionals play an essential role both in psychoeducational counseling, awareness and prevention initiatives, and in the early detection of cannabis use and the diagnosis and treatment of cannabis abuse and dependence. It is important for these professionals to have up-to-date knowledge on early exposure in order to inform the patients about the possible consequences, so that they can develop the necessary interventions in susceptible populations [66].

## Conclusions

The use of cannabis during pregnancy could produce neurochemical alterations both in humans and in research animals. From a clinical perspective, medical and psychiatric alterations have been described both in cross-sectional and in cohort studies.

Althoght there is some controversial findings, some regional differences and there are methodological limitations of the studies, early detection is fundamental. It is also important to warn women about the risks of using cannabis during pregnancy in order to minimize the possible consequences, which mainly include affective disorders and ADHD and which depend on duration and intensity of the prenatal exposure. In this regard, awareness campaigns may be an essential tool.

An active involvement is required from primary care, obstetricians, pediatric, mental health and drug dependence services. Longitudinal prospective studies need to be designed to finally identify all the consequences of prenatal exposure to cannabis regarding developmental alterations, neuro-cognitive side effects and mental disorders among the exposed population.

## Data Availability

Data sharing not applicable to this article as no datasets were generated or analysed during the current study.

## Abbreviations

5HT: Serotonin
ADHD: Attention deficit hyperactivity disorder
CCK: Cholecystokinin
CNS: Central Nervous System
CRF: Corticotropin-releasing factor
DNA: Deoxyribonucleic acid
EDADES: Spain Household Survey on Alcohol and Drugs from the National Drugs Plan
EMCDDA: European Monitoring Centre for Drugs and Drug Addiction
ESPAD: European School Survey Project on Alcohol and Other Drugs
ESTUDES: Secondary School Survey on Drugs in Spain
RNA: Ribonucleic acid
RNAm: Messenger ribonucleic acid
THC: Tetrahydrocannabinol
